# Effects of Atrazine on Estrogen Receptor** α**– and G Protein–Coupled Receptor 30–Mediated Signaling and Proliferation in Cancer Cells and Cancer-Associated Fibroblasts

**DOI:** 10.1289/ehp.1408586

**Published:** 2015-01-16

**Authors:** Lidia Albanito, Rosamaria Lappano, Antonio Madeo, Adele Chimento, Eric R. Prossnitz, Anna Rita Cappello, Vincenza Dolce, Sergio Abonante, Vincenzo Pezzi, Marcello Maggiolini

**Affiliations:** 1 Department of Pharmacy, Health and Nutritional Sciences, University of Calabria, Rende, Italy; 2 Department of Internal Medicine, and; 3 UNM Cancer Center, UNM Health Sciences Center, University of New Mexico, Albuquerque, New Mexico, USA; * These authors contributed equally to this work.

## Abstract

Background: The pesticide atrazine does not bind to or activate the classical estrogen receptor (ER), but it up-regulates the aromatase activity in estrogen-sensitive tumor cells. The G protein estrogen receptor (GPR30/GPER) has been reported to be involved in certain biological responses to endogenous estrogens and environmental compounds exerting estrogen-like activity.

Objectives: We aimed to evaluate the potential of atrazine to trigger GPER-mediated signaling in cancer cells and cancer-associated fibroblasts (CAFs).

Methods and Results: Using gene reporter assays in diverse types of cancer cells, we found that atrazine did not transactivate endogenous ERα or chimeric proteins that encode the ERα and ERβ hormone binding domains. Conversely, atrazine was able to bind to GPER to induce ERK activation and the expression of estrogen target genes, which, interestingly, appeared to rely on both GPER and ERα expression. As a biological counterpart, atrazine stimulated the proliferation of ovarian cancer cells that depend on GPER and ERα, as evidenced by gene silencing experiments and the use of specific signaling inhibitors. Of note, through GPER, atrazine elicited ERK phosphorylation, gene expression, and migration in CAFs, thus extending its stimulatory role to these main players of the tumor microenvironment.

Conclusions: Our results suggest a novel mechanism through which atrazine may exert relevant biological effects in cancer cells and CAFs. On the basis of our data, atrazine should be included among the environmental contaminants that may elicit estrogenic activity through GPER-mediated signaling.

Citation: Albanito L, Lappano R, Madeo A, Chimento A, Prossnitz ER, Capello AR, Dolce V, Abonante S, Pezzi V, Maggiolini M. 2015. Effects of atrazine on estrogen receptor α– and G protein–coupled receptor 30–mediated signaling and proliferation in cancer cells and cancer-associated fibroblasts. Environ Health Perspect 123:493–499; http://dx.doi.org/10.1289/ehp.1408586

## Introduction

Atrazine belongs to the 2-chloro-*s*-triazine family of herbicides and is one the most common pesticide contaminants of groundwater and surface water (Miller et al. 2000). People who work in agriculture or reside near agricultural fields may have higher levels of exposure to atrazine through spray drift than the general population (Gammon et al. 2005). Occupational exposure to atrazine may occur during manufacturing, formulation operations, and application, whereas nonoccupational exposure might arise from drinking water or diet. Gammon et al. (2005) found absorbed dosage values that ranged from 1.8 to 6.1 μg/kg/day. Moreover, atrazine was not removed from the body within 24 hr; its metabolites were detected in urine 48 hr after an oral dose (Davidson 1988). Therefore, pathophysiological effects may occur after repeated dosing and result from an accumulation above a critical threshold.

Epidemiologic studies have associated long-term exposure to triazine herbicides with increased risk of ovarian cancer in female farm workers in Italy (Donna et al.1989) and breast cancer in the general population of Kentucky in the United States (Kettles et al. 1997). In addition, atrazine leads to tumor development in the mammary gland and reproductive organs of female F344 rats (Pintér et al. 1990). Given the potential ability of atrazine to interfere with reproduction and to cause cancer, the European Union banned its use (Sass and Colangelo 2006). However, the U.S. Environmental Protection Agency has approved the use of atrazine due to a lack of a clear association between the levels of exposure and cancer incidence in pesticide applicators (Sass and Colangelo 2006).

Previous studies have demonstrated that triazine herbicides are not able to bind to or activate the classical estrogen receptor (ER) (Connor et al. 1996; Tennant et al. 1994). In recent years, increasing evidence has shown that steroid hormones, including estrogens, can rapidly interact with receptors located within or near the cell membrane (Norman et al. 2004). Moreover, Thomas (2000) suggested that nongenomic estrogen actions, like genomic ones, may be triggered by environmental estrogens. Of note, these compounds compete with estradiol–peroxidase conjugate for binding to estrogen membrane receptors and exert agonist effects through diverse transduction pathways in different cell contexts (Nadal et al. 2000). In addition, a seven-transmembrane receptor, namely GPR30/GPER (G protein estrogen receptor), has been shown to mediate relevant biological responses to estrogens (Maggiolini and Picard 2010). In this regard, our study and others have demonstrated that GPER is involved in multiple actions triggered by estrogenic compounds, including environmental contaminants, in a variety of cancer cells as well as in cancer-associated fibroblasts (CAFs) (Albanito et al. 2007; Lappano et al. 2010; Madeo and Maggiolini 2010; Pandey et al. 2009; Pupo et al. 2012; Vivacqua et al. 2006a, 2006b).

In the present study, we demonstrate that gene expression changes and growth effects induced by atrazine in ovarian cancer cells rely on both GPER and ERα. Furthermore, we show that GPER alone is able to mediate the stimulatory effects exerted by atrazine in ER-negative SkBr2 breast cancer cells and CAFs.

## Materials and Methods

*Reagents*. We purchased atrazine [2-chloro-4-(ethylamine)-6-(isopropylamine)-*s*-triazine], 17β-estradiol (E2), *N*-[2-(*p*-bromocinnamylamino)ethyl]-5-isoquinolinesulfonamide dihydrochloride (H89), wortmannin (WM), and PD98059 (PD) from Sigma-Aldrich (Milan, Italy); AG1478 (AG) and 1-*tert*-butyl-3-(4-chlorophenyl)-1H-pyrazolo[3,4-*d*]pyrimidin-4-amine (PP2) from Biomol Research Laboratories (DBA, Milan, Italy); ICI 182,780 (ICI) from Tocris Chemicals (Bristol, UK); and GF109203X (GFX) from Calbiochem (VWR International, Milan, Italy). All compounds were solubilized in DMSO except E2 and PD, which were dissolved in ethanol.

*Cell culture*. Human BG-1 and 2008 ovarian cancer cells as well as human Ishikawa endometrial cancer cells were kindly provided by D. Picard (University of Geneva, Geneva, Switzerland) and were maintained in Dulbecco’s modified Eagle medium (DMEM) without phenol red and supplemented with 10% fetal bovine serum (FBS) and 100 μg/mL penicillin/streptomycin. Human MCF-7 and SkBr2 breast cancer cells were maintained in DMEM with phenol red and RPMI 1640 without phenol red, respectively, supplemented with 10% FBS and antibiotics. CAFs were extracted as previously described (Madeo and Maggiolini 2010). Briefly, breast cancer specimens were collected from primary tumors of patients who had undergone surgery. Signed informed consent was obtained from all patients and from the institutional review board of the Regional Hospital of Cosenza. Tissues from tumors were cut into smaller pieces (1–2 mm diameter), placed in digestion solution (400 IU collagenase, 100 IU hyaluronidase, and 10% serum, containing antibiotic and antimycotic solution), and incubated overnight at 37°C. Cells were then separated by differential centrifugation at 90 ×*g* for 2 min. Supernatant containing fibroblasts was centrifuged at 485 ×*g* for 8 min; the pellet obtained was suspended in fibroblast growth medium (Medium 199 and Ham’s F12 mixed 1:1, supplemented with 10% FBS and antibiotics) and cultured at 37°C in 5% CO_2_. Primary cell cultures of breast fibroblasts were characterized using immunofluorescence (data not shown) as described previously (Pupo et al. 2012).

Cells were switched to medium without serum the day before immunoblots and reverse-transcription polymerase chain reaction (RT-PCR) experiments.

*Plasmids and luciferase assays*. We used the firefly luciferase reporter plasmids ERE-luc for ERα (Bunone et al. 1996) and GK1 for the Gal4 fusion proteins (Gal-ERα and Gal-ERβ) (Seipel et al. 1992; Webb et al. 1998). The Renilla luciferase expression vector pRL-TK (Promega, Milan, Italy) was used as a transfection standard. On the day before transfection, cells (1 × 10^5^) were plated into 24-well dishes in regular medium, which was replaced with medium supplemented with 1% charcoal-stripped FBS lacking phenol red on the day of transfection. Transfections were performed using X-tremeGene 9 reagent (Roche Diagnostics, Milan, Italy), as recommended by the manufacturer with a mixture containing 0.3 μg of reporter plasmid, 1 ng of pRL-TK, and 0.1 μg of effector plasmid where applicable. After 5–6 hr, ligands were added and cells were incubated for 16–18 hr. Luciferase activity was measured with the Dual Luciferase kit (Promega Italia, Milan, Italy) according to the manufacturer’s recommendations. Firefly luciferase values were normalized to the internal transfection control (pRL-TK). Normalized relative light unit values obtained from cells treated with vehicle (0.001% ethanol in medium) were set as 1-fold induction, upon which the activity induced by treatments was calculated.

*Gene silencing experiments*. Cells were plated onto 10-cm dishes and transfected for 24 hr before treatments using X-tremeGene 9 reagent (Roche Diagnostics). We purchased ERα small interfering RNA (siRNA) and the respective control from Sigma-Aldrich. The short hairpin RNA (shRNA) constructs used to knock down the expression of GPER and CTGF (connective tissue growth factor) and the unrelated shRNA control constructs have been described previously (Pandey et al. 2009).

*RT-PCR*. Gene expression was evaluated by semiquantitative RT-PCR as previously described (Maggiolini et al. 1999). Briefly, quantitative RT-PCR involved direct incorporation of digoxigenin-11-dUTP (DIG-dUTP) during amplification of cDNAs, separation of RT-PCR products by agarose gel electrophoresis, Southern transfer to a nylon membrane, and chemiluminescent detection with an anti-DIG antibody. The sequences of primers used are provided in Supplemental Material, Table S1.

*Western blotting*. Cells were grown in 10-cm dishes, exposed to ligands, and then lysed as previously described (Pandey et al. 2009). Protein concentrations were determined using Bradford reagent (Sigma-Aldrich) according to the manufacturer’s recommendations. Equal amounts of whole protein extract were resolved on a 10% SDS-polyacrylamide gel and transferred to a nitrocellulose membrane (Amersham Biosciences, GE Healthcare, Milan, Italy). Membranes were probed overnight at 4°C with antibodies against ERα (F-10, catalog no. sc-8002), GPER (N-15, catalog no. sc-48525-R), c-fos (H-125, catalog no. sc-7202), CTGF (L-20, catalog no. sc-14939), β-actin (AC-15, catalog no. sc-69879), phosphorylated ERK1/2 (E-4, catalog no. sc-7383), and ERK2 (C-14; catalog no. sc-154) (Santa Cruz Biotechnology, DBA, Milan, Italy), and then revealed using the ECL System from GE Healthcare (Milan, Italy).

*c-Src kinase assay*. Cell lysates were incubated with approximately 1 μg/mL mouse monoclonal anti-c-Src antibody (clone 327, catalog no. ab16885; Abcam, Prodotti Gianni S.r.L., Milan, Italy) overnight at 4°C, then added to an equal amount of goat anti-mouse IgG antibody and incubated for an additional 30 min. After 40 μL of a 50% suspension of protein G Sepharose was added, incubation continued for 30 min. The samples were centrifuged; the pellets were washed four times with 1 mL lysis buffer and then used for c-Src kinase assays. The activity of c-Src kinase was assayed using acidiﬁed enolase (0.5 mg/mL) as a substrate (Di Domenico et al. 1996).

*Ligand binding assay*. SkBr2 cells were grown in 10-cm cell culture dishes and incubated with 50 nM [2,4,6,7-3H]E2 (89 Ci/mmol; GE Healthcare) in the presence or absence of increasing concentrations of nonlabeled E2 or atrazine for 2 hr at 37°C. Cells were then washed with ice-cold phosphate-buffered saline (PBS); after 100% ethanol extraction of cells, radioactivity was measured by liquid scintillation counting. The displacement of [^3^H]E2 binding by the competitors was expressed as a percentage of the maximum specific binding of E2.

*Proliferation assay*. Cells were seeded in 24-well plates in regular growth medium. After cells attached, they were incubated in medium containing 2.5% charcoal-stripped FBS, transfected for 24 hr, and treated as indicated, with transfection and treatments renewed every 2 days. Cells were counted using an automated cell counter (Life Technologies, Monza, Italy) following the manufacturer’s recommendations.

*Cell cycle analysis*. Cells synchronized for 24 hr in serum-free medium were transfected, treated for 8 hr, and subjected to fluorescence-activated cell sorting (FACS) analysis. Adherent and floating cells were centrifuged and resuspended in PBS containing 20 μg/mL propidium iodide plus 40 μg/mL ribonuclease (Sigma-Aldrich) for 1 hr. Cells were then subjected to FACS analysis (FACS Jazz, BD, Milan, Italy) and results were expressed in terms of percentage.

*Transwell cell migration assay*. The migration assay was performed in CAFs using Boyden chambers (Costar Transwell, 8 mm poly-carbonate membrane; Sigma-Aldrich). For knockdown experiments, cells were transfected for 24 hr, then seeded in the upper chambers. Treatments were added to the serum-free medium in the bottom wells. After 24 hr, cells on the bottom of the membrane were fixed, stained with Giemsa (Sigma-Aldrich), photographed, and counted.

*Statistical analysis*. Statistical analysis was performed using analysis of variance followed by Newman–Keuls testing to determine differences in means. We considered *p* < 0.05 to be statistically significant.

## Results

**Figure 1:**
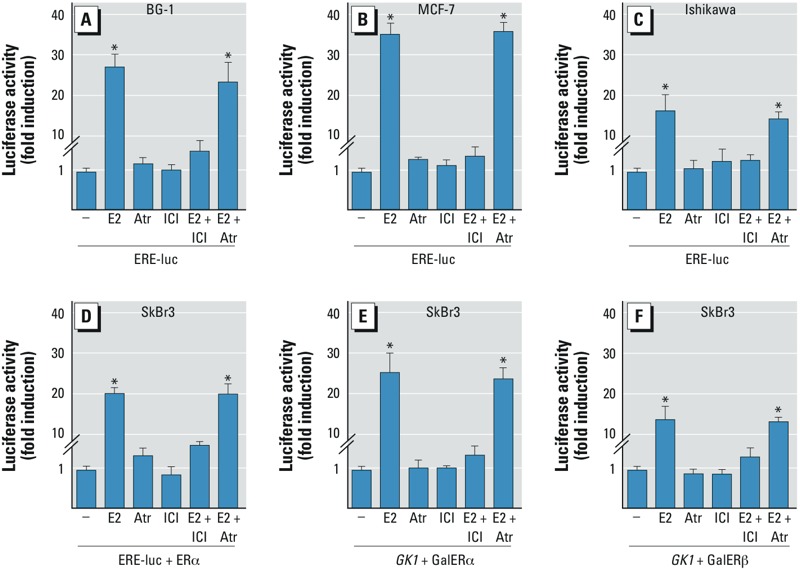
Luciferase activity in cells transfected with a luciferase reporter plasmid and treated with vehicle (–), 100 nmol/L E2, 1 μmol/L atrazine (Atr), 10 μmol/L ICI (ER antagonist), E2 + ICI, or Atr + ICI. ERα transactivation in BG-1 (*A*), MCF-7 (*B*), or Ishikawa (*C*) cells transfected with ERE-luc before treatment. (*D–F*) SkBr3 cells transfected with ERE-luc and ERα expression plasmid (*D*), Gal4 reporter gene (*GK1*) plus the Gal4 fusion proteins encoding the hormone-binding domain of ERα (GalERα; *E*), or *GK1* plus GalERβ (*F*) before treatment. Luciferase activity was normalized to the internal transfection control, and values for vehicle controls were set as 1-fold induction. Values shown are mean ± SD of three independent experiments performed in triplicate. *p < 0.05 compared with vehicle treatment.

*ER*α *and ER*β *activation*. On the basis of evidence that atrazine influences the development of estrogen-sensitive tumors (Cooper et al. 2007), we first evaluated whether atrazine could activate a transiently transfected ER reporter gene in estrogen-sensitive ovarian BG-1, breast MCF-7, and endometrial Ishikawa cancer cells. E2 treatment induced a strong ERα transactivation, which was prevented using the ER antagonist ICI (Figure 1A–C). In contrast, atrazine failed to stimulate luciferase expression or to block the activation of ERα by E2 (Figure 1A–C). Likewise, an expression vector encoding ERα transfected in ER-negative SkBr2 breast cancer cells was not activated by atrazine (Figure 1D). To confirm that atrazine does not act as an ERα agonist and to examine whether ERβ could respond to atrazine, we turned to a heterologous system. Chimeric proteins consisting of the DNA binding domain of the yeast transcription factor Gal4 and the ERα or ERβ hormone binding domain, which were transiently transfected in SkBr2 cells, showed a strong transactivation by E2 but not by atrazine (Figure 1E,F), confirming that atrazine did not transactivate ER.

**Figure 2:**
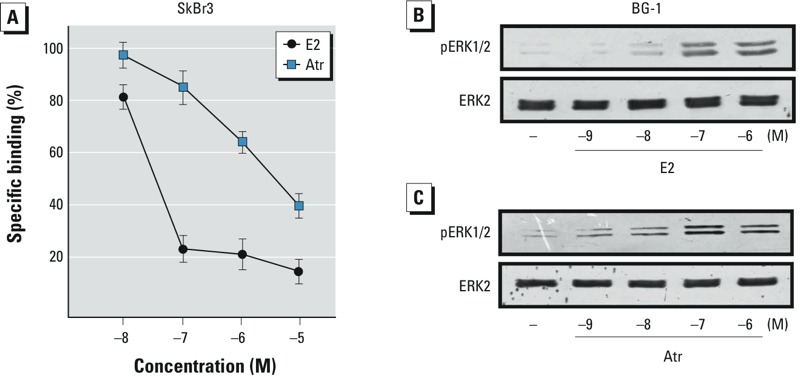
(*A*) Ligand binding assay for GPER in SkBr3 cells exposed to increasing concentrations of E2 or atrazine (Atr) for 2 hr. Competition curves of unlabeled E2 and Atr are expressed as a percentage of maximum specific [^3^H]E2 binding; each data point represents the mean ± SD of three separate experiments performed in triplicate. (*B,C*) ERK1/2 phosphorylation in BG-1 cells exposed to increasing concentrations of E2 (*B*) or Atr (*C*) for 20 min; ERK2 served as a loading control. Data shown are representative of three independent experiments.

*GPER binding and ERK phosphorylation*. Considering that diverse environmental contaminants exhibit binding affinity for GPER (Thomas and Dong 2006), we performed ligand-binding studies using radiolabeled E2 as a tracer in ER-negative and GPER-positive SkBr2 breast cancer cells (Lappano et al. 2010, 2012a, 2012b). Atrazine displaced the tritiated E2 in a dose-dependent manner (Figure 2A), demonstrating the ability of atrazine to bind to GPER, although with a lower binding affinity compared with E2.

**Figure 3:**
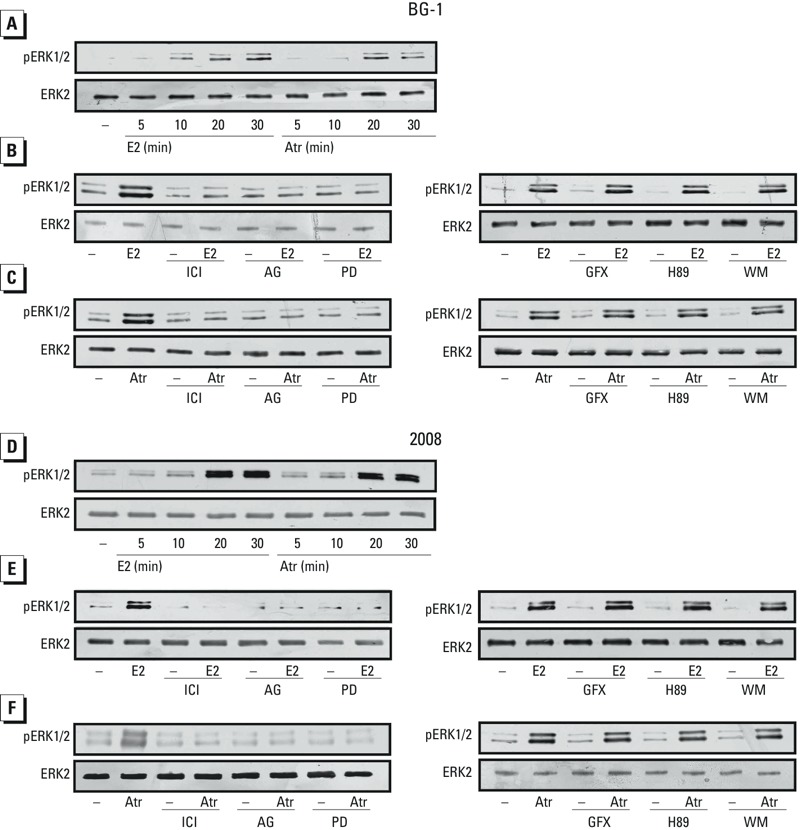
ERK1/2 phosphorylation in BG-1 (*A*) and 2008 (*D*) cells treated with vehicle (–), 100 nmol/L E2, or 1 μmol/L atrazine (Atr) for up to 30 min. ERK1/2 phosphorylation in BG-1 (*B*,*C*) and 2008 (*E*,*F*) cells treated for 20 min with vehicle, 100 nmol/L E2, or 1 μmol/L Atr, alone and in combination with 10 μmol/L ICI (ER antagonist), 10 μmol/L AG (EGFR inhibitor), 10 μmol/L PD (MEK kinase inhibitor), 10 μmol/L GFX (protein kinase C inhibitor), 10 μmol/L H89 (protein kinase A inhibitor), or 10 μmol/L WM (phosphoinositide 3-kinase inhibitor). ERK2 served as a loading control. Data shown are representative of three independent experiments.

Several studies have recently demonstrated that estrogens and xenoestrogens can generate rapid signaling via second messengers such as Ca^2+^, cAMP, nitric oxide, and G proteins, which in turn activate numerous downstream kinases (Bulayeva and Watson 2004; Lappano and Maggiolini 2011). In this regard, we found that atrazine stimulated ERK phosphorylation in BG-1 cells, in a manner similar to E2 (Figure 2B,C). Hence, we performed time-course experiments using specific pharmacological inhibitors in BG-1 and 2008 ovarian cancer cells that exhibit a similar receptor expression pattern (Safaei et al. 2005). As shown in Figure 3A and D, E2 and atrazine induced ERK phosphorylation in a time-dependent manner in both ovarian cancer cell lines. The treatment with the ER antagonist ICI, the EGFR inhibitor AG, and the ERK inhibitor PD prevented ERK activation upon exposure to E2 and atrazine, whereas GFX, H89, and WM, inhibitors of protein kinase C (PKC), protein kinase A (PKA) and phosphoinositide 3-kinase (PI3K), respectively, did not (Figure 3B,C,E,F). Taken together, the inhibitory effects elicited by ICI, AG, and PD suggest that the EGFR/ERK transduction pathway and ERα are involved in ERK activation induced by E2 and atrazine.

*mRNA expression of estrogen target genes*. Using BG-1 cells, we evaluated the ability of atrazine to regulate the expression of genes that have been shown to respond to estrogens and environmental contaminants (Pandey et al. 2009; Pupo et al. 2012). To this end, we performed semiquantitative RT-PCR experiments and compared standardized mRNA levels with a housekeeping gene encoding the ribosomal protein 36B4. After 1 hr treatment with atrazine, *c-fos*, *CTGF*, and cyclin A levels were enhanced, although to a lesser extent than after E2 treatment. E2 also stimulated the expression of *PR* (progesterone receptor), *pS2*, and cyclin D1 (see Supplemental Material, Table S2). After 24-hr treatment with atrazine, *PR*, *pS2*, and cyclin A levels were increased, whereas E2 induced not only the expression of these genes but also the expression of *c-fos*, cathepsin D, cyclin D1, and cyclin E (see Supplemental Material, Table S2). Similar results were obtained in 2008 cells (data not shown). Migliaccio et al. (2007) reported that estrogens may signal through intracellular effectors such as c-Src, which in turn activate complex transduction networks leading to gene expression changes in cancer cells. Hence, we used the c-Src inhibitor PP2 to evaluate the involvement of c-Src in gene transcription stimulated by atrazine and E2 in BG-1 cells (see Supplemental Material, Table S3) and 2008 cells (data not shown). The up-regulation of *PR*, *pS2*, and cyclin A induced by E2 was decreased in the presence of PP2, although the induction of these genes still remained statistically significant. In contrast, the gene transcription prompted by atrazine was not influenced by PP2. In accordance with these findings, atrazine did not enhance c-Src kinase activity on enolase as determined in BG-1 cell lysates immunoprecipitated with anti-c-Src antibody (data not shown).

*Transduction pathways involved in the up-regulation of c-fos protein levels*. Using c-fos expression as a molecular sensor of atrazine action, we sought to determine whether atrazine could also regulate c-fos at a protein level as well as the transduction pathways involved in this response. Interestingly, the up-regulation of c-fos observed in BG-1 and 2008 cells treated for 2 hr with E2 (see Supplemental Material, Figure S1A,C) or atrazine (see Supplemental Material, Figure S1B,D) was abolished after treatment with the ER antagonist ICI, the EGFR inhibitor AG, or the ERK inhibitor PD (see Supplemental Material, Figure S1). On the contrary, GFX, H89, and WM—inhibitors of PKC, PKA, and PI3K, respectively—did not interfere with c-fos stimulation (see Supplemental Material, Figure S1). These results in ovarian cancer cells suggest that atrazine triggered an increase of c-fos protein through ERα and the EGFR–MAPK transduction pathway, thus confirming the results obtained in the ERK activation studies. On the basis of these data and our previous results showing that c-fos stimulation by E2 occurs through GPER and ERα in cancer cells expressing both receptors (Albanito et al. 2007), we examined whether atrazine could act in a similar manner. Silencing ERα (see Supplemental Material, Figure S2A,B,E,F) or GPER (see Supplemental Material, Figure S2C,D,G,H) in BG-1 and 2008 cells indicated that E2 and atrazine were not able to induce the expression of *c-fos* or *CTGF*, a main GPER-target gene (see Supplemental Material, Figure S2). Next, to evaluate whether atrazine could induce a rapid response in an ER-negative and GPER-positive cell context, we used SkBr2 breast cancer cells. The ERK phosphorylation and the induction of c-fos and CTGF we observed after stimulating cells with atrazine (see Supplemental Material, Figure S3) or E2 (data not shown) were abolished, knocking down the expression of GPER, similar to our previous data obtained using estrogens (Lappano et al. 2010, 2012a, 2012b; Maggiolini et al. 2004).

**Figure 4:**
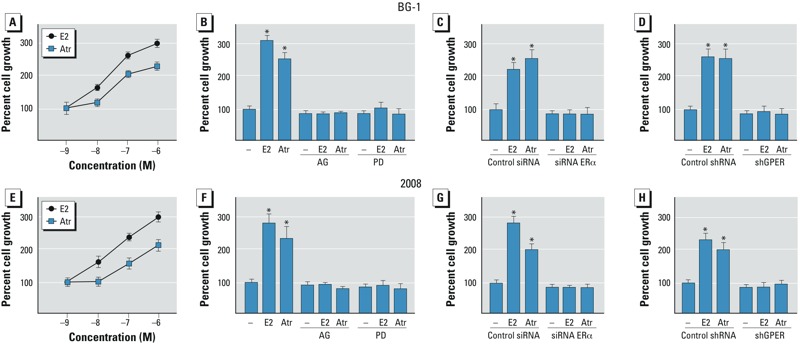
Proliferation of BG-1 (*A–D*) and 2008 (*E–H*) cells treated with E2 or atrazine (Atr). Proliferation of BG-1 (*A*) and 2008 (*E*) cells in response to increasing concentrations of E2 or Atr. Proliferation of BG-1 (*B*–*D*) and 2008 (*F*–*H*) cells treated with vehicle (–), 100 nmol/L E2, or 1 μmol/L Atr alone or in combination with 10 μmol/L AG or 10 μmol/L PD (*B*,*F*), or transfected with control siRNA or siRNA ERα (*C*,*G*) or control shRNA or shGPER (*D*,*H*). Proliferation of vehicle-treated cells was set at 100%; Values shown are mean ± SD of three independent experiments performed in triplicate. *p < 0.05 compared with vehicle-treated cells.

*BG-1 and 2008 cell proliferation and cell cycle analysis*. We found that E2 and atrazine induced the proliferation of BG-1 and 2008 cells in a concentration-dependent manner (Figure 4A,E). The growth effects elicited by E2 and atrazine were not evident in the presence of AG and PD (Figure 4B,F) or when the expression of GPER or ERα was silenced (Figure 4C,D,G,H), suggesting that both receptors, along with the EGFR/MAPK transduction pathway, contribute to the proliferation induced by atrazine. To further corroborate these results, we performed cell cycle analysis and found that the increase of BG-1 cells in G_2_/M phase induced by atrazine was absent when the expression of ERα or GPER was silenced (see Supplemental Material, Figure S4).

**Figure 5:**
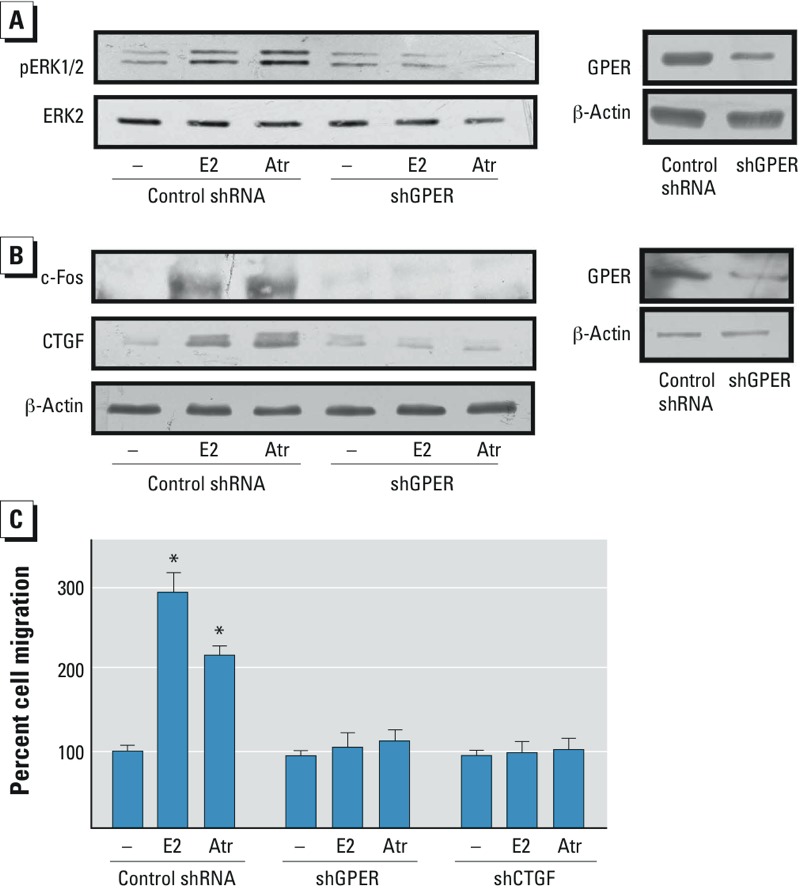
ERK phosphorylation, gene expression changes, and cell migration in CAFs treated with vehicle (–), 1 nmol/L E2, or 1 μmol/L atrazine (Atr). ERK1/2 phosphorylation (*A*) and c-fos and CTGF expression (*B*) in CAFs silenced for GPER expression. The efficacy of GPER silencing was ascertained by immunoblots (right). ERK2 and β-actin served as loading controls; data shown are representative of three independent experiments. (*C*) Migration of CAFs in cells transfected with shGPER or shCTGF before treatment. Migration of vehicle-treated cells was set at 100%; values shown are mean ± SD of three independent experiments performed in triplicate. *p < 0.05 compared with vehicle-treated cells.

*ERK phosphorylation, gene expression changes, and migration in CAFs*. To provide further evidence regarding the ability of atrazine to trigger biological responses through GPER, we evaluated the activity of atrazine in CAFs obtained from breast tumor patients (Madeo and Maggiolini 2010). In these cells that express GPER and lack ERα (Figure 5; see also Supplemental Material, Figure S5), E2 and atrazine stimulated ERK phosphorylation and the expression of both c-fos and CTGF through GPER (Figure 5A,B). In addition, the migration of CAFs promoted by E2 and atrazine was abolished, knocking down the expression of GPER or CTGF (Figure 5C), which exerts an acknowledged role in the migration of cancer cells (Pandey et al. 2009). Collectively, these results indicate that atrazine induced relevant biological effects through GPER in CAFs, cells that contribute to the progression of cancer by acting as key players within the tumor microenvironment (Bhowmick et al. 2004).

## Discussion

In the present study, we observed that atrazine exerted estrogen-like activity in ovarian and breast cancer cells and in CAFs through GPER, which mediated estrogen signals, as reported in previous studies (reviewed by Prossnitz and Barton 2014).

Previous studies have reported that atrazine elicited estrogen action by up-regulating aromatase activity in certain cancer cells with elevated aromatase levels (Sanderson et al. 2001) but not by binding to or activating ERα (Connor et al. 1996; Roberge et al. 2004; Tennant et al 1994). Using different model systems, we confirmed that atrazine did not activate ERα although it did induce the expression of several estrogen target genes. However, in a previous study, Dudek and Picard (2008) reported that distinct compounds and factors recruited ERα to gene promoter sequences different from the classical estrogen responsive elements. Furthermore, our data show that GPER and ERα, along with the EGFR/MAPK pathway, contribute to the biological responses to atrazine in diverse cancer cells. Thus, our findings indicate that a complex interplay between different estrogen receptors and transduction pathways contributes to atrazine activity, which may be still evident in cell contexts where only GPER is expressed, such as SkBr2 cells and CAFs.

Results of the present study corroborate our previous data regarding the physical interaction between GPER and ERα and the biological responses triggered by the functional crosstalk of these receptors (Vivacqua et al. 2009). Previous research (Migliaccio et al. 2006; Vivacqua et al. 2009) showed that EGFR co-immunoprecipitates with ERα and GPER, suggesting that an intricate crosstalk may occur among these main transduction mediators in cancer cells. Collectively, these findings indicate that estrogens and estrogen-like compounds may exert pleiotropic actions through ERα in a direct manner, as well as via GPER–EGFR transduction signaling, which may engage ERα toward the stimulation of cancer cells. In accordance with these observations, in BG-1 and 2008 cancer cells the silencing of GPER or ERα expression and the inhibition of the EGFR/ERK signaling prevented the action of atrazine, confirming that a cooperation between these receptors is involved in the biological responses to atrazine. In addition, the present data are in accordance with previous studies showing that xenoestrogens may mimic estrogen action in several animal and cell models (Bulayeva and Watson 2004; Nadal et al. 2000).

A subset of estrogen-sensitive cell tumors can proliferate regardless of ER expression. Under these conditions, which may be represented by SkBr2 breast cancer cells, GPER/EGFR signaling could allow stimulatory effects by environmental estrogens, as shown in the present study and in previous studies (Maggiolini and Picard 2010; Pupo et al. 2012). Thus, multiple transduction pathways triggered at the membrane level, as well as within the diverse cell types, contribute to the nature and the magnitude of biological responses to distinct estrogenic compounds. Consequently, these agents should be examined to determine the complex mechanistic and functional outcomes that result from an interaction with a repertoire of different receptor proteins.

Here, we have provided novel insights regarding the potential role of GPER in mediating the action of atrazine, not only in estrogen-sensitive tumors but also in CAFs. Because CAFs play a key role within the tumor microenvironment as well as at metastatic sites (Bhowmick et al. 2004), our results further extend the knowledge of molecular mechanisms through which atrazine may contribute to cancer progression. Future studies are needed to evaluate the effects exerted by atrazine *in vivo* through GPER in cancer progression and other pathophysiological conditions.

## Supplemental Material

(4.4 MB) PDF
