# Supplementary material for: Effects of Atrazine on Estrogen Receptor α– and G Protein–Coupled Receptor 30–Mediated Signaling and Proliferation in Cancer Cells and Cancer-Associated Fibroblasts
Source: Environ Health Perspect. 2015 Jan 16;123(5):493–9. doi: 10.1289/ehp.1408586 (PMC4421771; doi:10.1289/ehp.1408586)
Supplement: (4.4 MB) PDF [file ehp.1408586.s001.508.pdf]

Environmental Health Perspectives doi:10.1289/ehp.1408586

Supplemental Material

Effects of Atrazine on Estrogen Receptor α– and G Protein–Coupled Receptor 30–Mediated Signaling and Proliferation in Cancer Cells and Cancer-Associated Fibroblasts

Lidia Albanito, Rosamaria Lappano, Antonio Madeo, Adele Chimento, Eric R. Prossnitz, Anna Rita Cappello, Vincenza Dolce, Sergio Abonante, Vincenzo Pezzi, and Marcello Maggiolini

Table S1. Sequences of primers used for RT-PCR.

Gene	Primer Fw	Primer Rv	Yield products (bp)
c-fos	AGAAAAGGAGAATCCGAAGGGAAA	ATGATGCTGGGACAGGAAGTC	345
CTGF	ATGGCATGAAGCCAGAGAGT	GGTCAGTGAGCACGCTAAAA	420
PR	ACACCTTGCCTGAAGTTTCG	CTGTCCTTTTCTGGGGGACT	196
pS2	TTCTATCCTAATACCATCGACG	TTTGAGTAGTCAAAGTCAGAGC	210
CathepsinD	AACAACAGGGTGGGCTTC	ATGCACGAAACAGATCTGTGCT	303
CyclinA	GCCATTAGTTTACCTGGACCCAGA	GCCATTAGTTTACCTGGACCCAGA	354
CyclinD1	TCTAAGATGAAGGAGACCATC	GCGGTAGTAGGACAGGAAGTTGTT	354
CyclinE	CCTGACTATTGTGTCCTGGC	CCCGCTGCTCTGCTTCTTAC	488
36B4	CTCAACATCTCCCCCTTCTC	CAAATCCCATATCCTCGTCC	408

Table S2. mRNA expression (% variation ± SD) as evaluated by densitometric analysis in BG-1 cells treated for 1 hr or 24 hr with 100 nmol/L E2 and 1 μmol/L atrazine (Atr).

Gene	E2 (1hr)	E2 (24hr)	Atr (1hr)	Atr (24hr)
c-fos	423 ± 28*	239 ± 17*	269 ± 21*	120 ± 9
CTGF	498 ± 22*	120 ± 19	320 ± 17*	125 ± 12
PR	228 ± 18*	298 ± 18*	122 ± 18	180 ± 11*
pS2	175 ± 17*	270 ± 21*	99 ± 19	187 ± 20*
CathepsinD	106 ± 9	217 ± 16*	102 ± 5	109 ± 6
CyclinA	262 ± 22*	293 ± 23*	220 ± 20*	190 ± 22*
CyclinD1	258 ± 19*	242 ± 19*	107 ± 4	118 ± 8
CyclinE	120 ± 11	343 ± 21*	118 ± 8	119 ± 10

Values in cells treated with vehicle were set as 100% and the expression induced by treatments is presented as percentage variation. The significant difference ($p<0.05$) of values obtained upon E2 or Atr treatments respect to vehicle was marked with an asterisk (*).

Table S3. mRNA expression (% variation ± SD) as evaluated by densitometric analysis in BG-1 cells treated for 1 hr or 24 hr with 100nmol/L E2 and 1μmol/L atrazine (Atr) alone and in combination with 1μmol/L c-Src inhibitor (PP2).

Gene	E2 (1 hr)	E2 + PP2 (1 hr)	E2 (24 hr)	E2 + PP2 (24 hr)	Atr (1 hr)	Atr + PP2 (1 hr)	Atr (24 hr)	Atr + PP2 (24 hr)
PR	203 ± 15*	152 ± 6**	251 ± 22*	180 ± 12**	110 ± 9	115 ± 11	172 ± 8*	188 ± 13*
pS2	182 ± 15*	139 ± 10**	290 ± 25*	205 ± 16**	105 ± 16	111 ± 10	168 ± 17*	170 ± 9*
CyclinA	247 ± 20*	183 ± 16**	275 ± 21*	210 ± 18**	215 ± 17*	210 ± 14*	178 ± 19*	161 ± 9*

Values in cells treated with vehicle were set as 100% and the expression induced by treatments is presented as percentage variation. The significant difference ($p<0.05$) of values obtained upon E2 or Atr treatments respect to vehicle was marked with an asterisk (*). The significant difference ($p<0.05$) of values obtained using E2 in combination with PP2 respect to E2 alone was marked with a double asterisk (**).

Figure S1

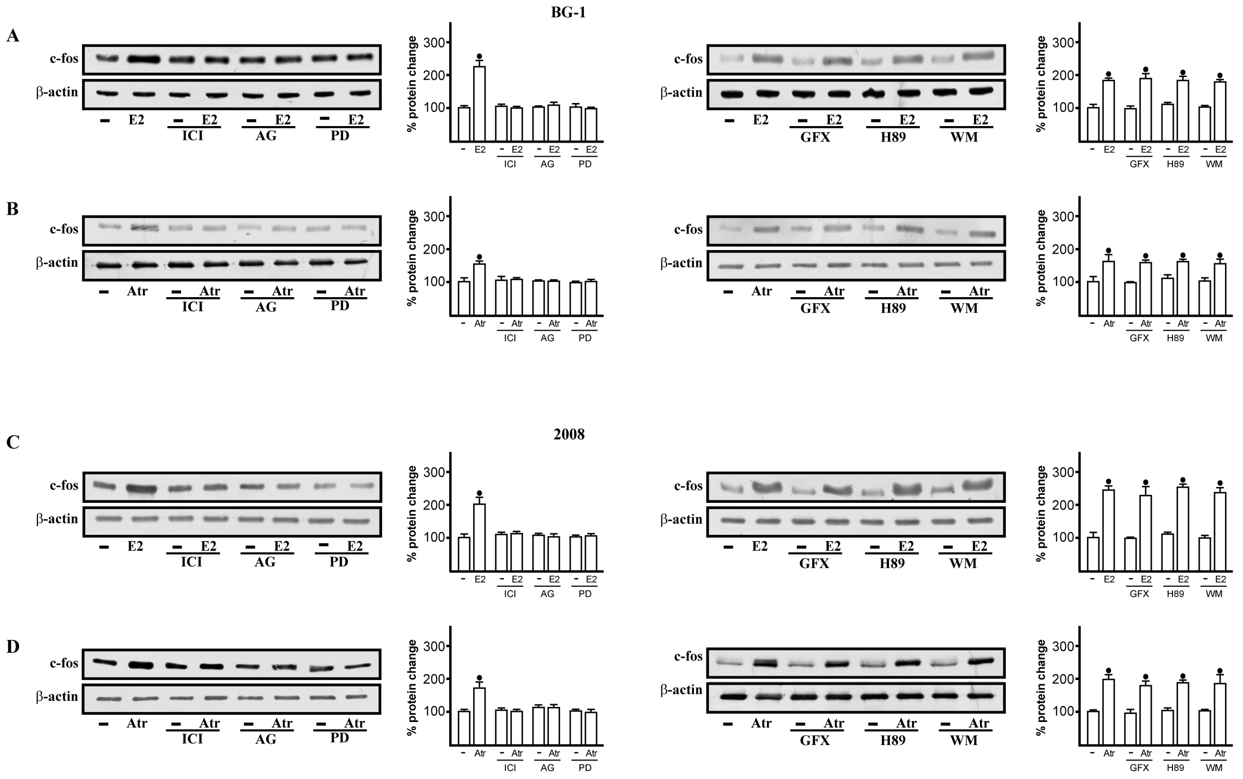

Figure S1. Immunoblots of c-fos from BG-1 (A,B) and 2008 (C,D) cells treated for 2hr as indicated with vehicle (–), 100nmol/L E2, 1µmol/L atrazine (Atr), 10µmol/L ICI, 10µmol/L AG, 10µmol/L PD, 10µmol/L GFX, 10µmol/L H89, 10µmol/L WM, which are inhibitors of ER, EGFR, MEK, PKC, PKA and PI3K, respectively. Side panels show densitometric analysis of the immunoblots. Data shown are representative of three independent experiments. Data obtained were normalized to β-actin. (•) indicates $p < 0.05$ for cells receiving vehicle (–) versus treatments.

Figure S2

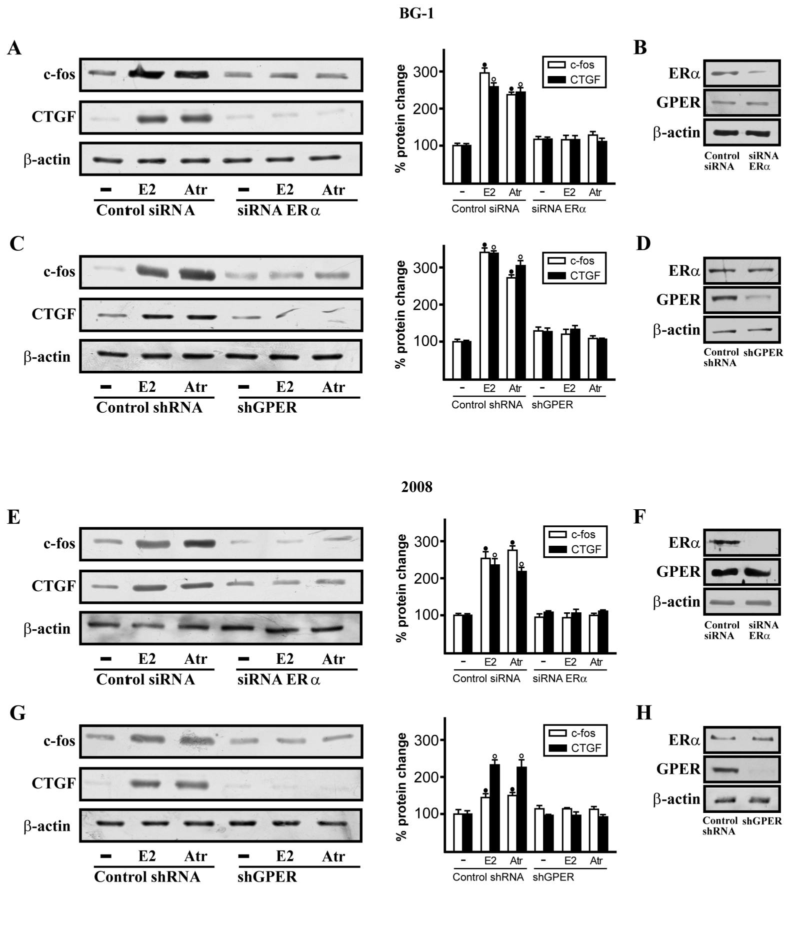

Supplemental Material, Figure S2

Figure S2. Immunoblots of c-fos and CTGF from BG-1 (A,C) and 2008 (E,G) cells silenced for ERα and GPER expression. Cells were transfected with control siRNA or siRNA-ERα (A,E), with control shRNA or shGPER (C,G) and then treated for 2hr with vehicle (–),100nmol/L E2 and 1μmol/L atrazine (Atr). Side panels show densitometric analysis of the immunoblots. Data shown are representative of three independent experiments. Data obtained were normalized to β-actin. The efficacy of ERα and GPER silencing was ascertained by immunoblots (B,D,F,H). Data obtained were normalized to β-actin. (•) and (○) indicate $p < 0.05$ for cells receiving vehicle (–) versus treatments.

Figure S3

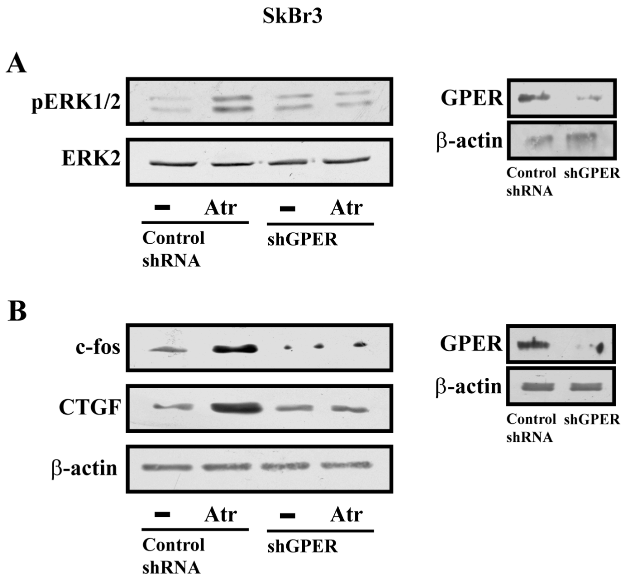

Figure S3. ERK1/2 phosphorylation (A) and c-fos and CTGF expression (B) silencing GPER expression in SkBr3 cells treated with vehicle (–) and 1 μmol/L atrazine (Atr). The efficacy of GPER silencing was ascertained by immunoblots, as shown in side panels. ERK2 and β-actin serve as loading controls. Data shown are representative of three independent experiments.

Figure S4

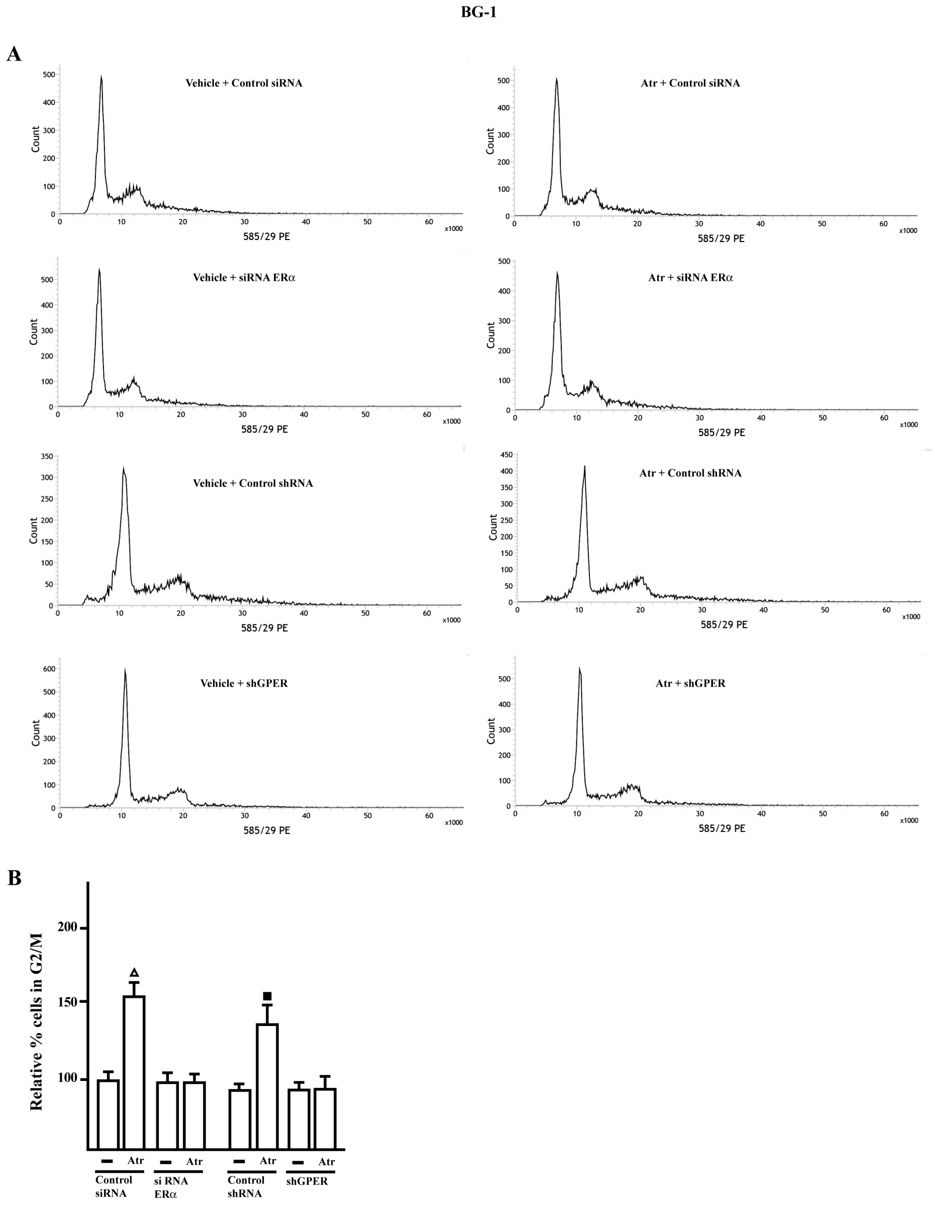

Figure S4. (A) BG-1 cells were transfected with control siRNA or siRNA ERα and with control shRNA or shGER, treated with vehicle (–) and 1 μmol/L atrazine (Atr) and then analyzed for the cell-cycle profile. Cell populations positive for propidium iodine staining were evaluated by FACS analysis. Panels show the cell cycle profile of a representative experiment. (B) The percentage of cells in G2/M phase is indicated, values are the mean ± SD of three independent experiments. (Δ, ▪) indicate $p < 0.05$ for cells receiving vehicle (–) versus treatments.

Figure S5

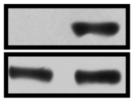

Figure S5. Western blot analysis of ERα protein expression in CAFs and MCF-7 breast cancer cells. Data shown are representative of three independent experiments. β-Actin antibody was used as loading control.
